# Reliability of a forensic odontology method for age-at-death estimation in adults: A Mexican case study

**DOI:** 10.1016/j.fsisyn.2024.100484

**Published:** 2024-06-26

**Authors:** Roberto Scendoni, Israel Soriano Vázquez, Isabella Lima Arrais Ribeiro, Stefano De Luca, Galina V. Zolotenkova, Serena Viva, Akiko Kumagai, Roberto Cameriere

**Affiliations:** a Department of Law, Institute of Legal Medicine (AgEstimation Project), University of Macerata, Macerata, Italy; b Extraordinary Mechanism for Human Identification (MEIF) – United Nations Population Fund (UNFPA), Mexico; c Postgraduate Program in Dentistry, Federal University of Paraíba, Campus I, João Pessoa, PB, 58051900, Brazil; d Área de Identificación Forense, Unidad de Derechos Humanos, Servicio Médico Legal, Santiago de Chile, Chile; e Department of Forensic Medicine, First Moscow State Medical University (Sechenov University), Moscow, Russia; f Department of Cultural Heritage, University of Salento, Lecce, Italy; g Division of Forensic Odontology and Disaster Oral Medicine, Department of Forensic Science, Iwate Medical University, Iwate, Japan; h Department of Medicine and Health Sciences, University of Molise, Campobasso, Italy

**Keywords:** Age estimation, Pulp/tooth area, Observer agreement, Forensic odontology, Human identification, Missing people

## Abstract

This study aimed to evaluate the reliability of an age estimation method based on the pulp⁄tooth area ratio by assessing intra- and inter-examiner agreement across five observers at different intervals. Using the same X-ray device and technical parameters, 96 digital periapical X-ray images of upper and lower canines were obtained from 28 deceased people in Central America, whose age at death ranged from 19 to 49 years. Excellent and good agreement of results were achieved, and there were no statistically significant differences. The R2 value for upper teeth (54.0%) was higher than the R2 value for lower teeth (45.7%). The highest intraclass correlation coefficient value was 0.995 (0.993–0.997) and the lowest 0.798 (0.545–0.895). Inter-examiner agreement was high with values of 0.975 (0.965–0.983) and 0.927 (0.879–0.955). This method is adequate for assessing age in missing and unidentified people, including victims of mass disasters.

## 1 Introduction

Age-at-death estimation in adults remains challenging due to the uncertainty involved in method selection and the challenge of making an optimal decision for a court of law, especially in criminal cases and mass disaster scenarios, where the skeletal remains are often commingled and incomplete [1].

The particular value of estimating age at death in forensic contexts has already been highlighted in several scientific publications [2]. However, forensic estimation of age at death faces issues of time and cost. An added complication is that it must often be performed on small body parts. One of the major aims of forensic experts is to achieve an accurate, non-destructive method of age-at-death estimation, with an acceptable margin of error, to ensure accurate reporting to law enforcement, and to narrow the list of missing persons for presumptive identification.

The Scientific Working Group for Forensic Anthropology (SWGANTH) states that the final age estimate is a matter of expert judgment by synthesizing all available information. Factors to be considered are: appropriateness of the reference data, skill in using one method over another, condition of the remains, applicability of statistical models, etc. [3]. In addition, the Organization of Scientific Area Committees for Forensic Science (OSAC), which strives to strengthen forensic standards, has published approved standards for age-at-death estimation [4]. The aim of these forensic standards is to provide the best available up-to-date information and guidance for estimating age based on human dentition. They include guidelines on obtaining forensic dental data and selecting the most appropriate recommended methodology to establish accurate assessments of chronological age in humans. They are intended for practitioners performing dental age assessments and for individuals, groups, or agencies that make use of the results. Age-at-death estimation assists in the identification of missing and unidentified individuals by narrowing search parameters. However, dental age assessment is based on large populations of identifiable human groups and involves an associated level of uncertainty, which needs to be quantified in the application of the method.

In a mass disaster involving a huge number of fatalities, it is necessary not only to handle the bodies with respect, but also to identify them so that families can find out what happened to their missing relatives and bury them quickly (a death certificate is needed in this case). Unidentified human remains should be deposited in registered places and properly identified, in accordance with regulations and protocols, such as the Minnesota Protocol [5], so that the protection of human remains can be guaranteed until their identification.

Today, Mexico faces a series of challenges in forensic practice: the so-called “War on Drugs” has resulted in multitude deaths and missing people as well as hundreds of illegal graves throughout the country and thousands of people internally displaced [6,7]. In addition, hundreds of thousands of Mexicans try to cross the Mexico–United States border annually and hundreds of these individuals die in the attempt [8]. Finally, due to its particular geographic position, surrounded by several volcanoes (including Popocatepetl Volcano, one of the most active stratovolcanoes in Mexico [9]), the country has been struck by several large earthquakes [10] and remains under constant risk of a mass disaster [11].

Although Mexican authorities have the duty to locate and identify victims, Mexico lacks an official protocol to regulate the systematic protection of bodies [12]. In addition, while much has been published worldwide on the most common methodologies for assessing a biological profile [13], publications from Mexico are limited [12].

Dental age estimation methods are based on biological changes over time that include growth and wear. The various approaches based on the formation and development of teeth involve the use of conventional radiographs, CT scans, or MRIs. Dental age estimation techniques are considered highly reliable in children and therefore useful in cases where it is important to distinguish between minor/major age. Over the years, methods have also been refined in adult subjects, resulting in reduced evaluation errors.

As indicated in Solheim [14], secondary dentine apposition in a tooth has a relatively high correlation with chronological age in adults and can be calculated using radiographic techniques. According to the last few works on this issue [15], once dental development is complete, rapid secondary dentine deposition is observed in the lower canine until the 25–30 years age group. In middle age, secondary dentine deposition slows down and is consistent. In the 6th decade of life, rapid dentine formation is once again observed.

In the last two decades, several studies have been published on age estimation using quantitative measurements of these morphological changes on X-ray images of teeth [16]. Cameriere et al. [17], who developed a method for assessing age based on pulp/tooth area ratio, found that canines showed the highest correlation with chronological age when compared with other types of teeth (e.g., incisors or premolars).

In such analyses, permanent canines are selected because they have long roots and distinct pulp margins and can therefore be easily measured in both panoramic and periapical X-rays [18]. In addition, they are single rooted and have the largest pulp areas among the monoradicular anterior teeth [19]. Furthermore, canines are more resistant and survive longer than any other teeth regardless of age, unaffected by the most common taphonomic and diagenetic changes (e.g., thermal alterations) [20]. The resistance of this particular tooth against most environmental alterations makes it a useful indicator for assessing age at death and for identifying victims of mass disasters. This last task is an intensive and demanding mission involving specialists from various disciplines.

Although new protocols have been developed to make the identification process faster and more accurate [4], each mass fatality incident results in new challenges for identification teams of well-trained experts. Since forensic personnel have to search for matching ante-mortem data within a specific age range from a missing persons list, the most precise age estimation technique should be selected in order to reduce estimation times and subjectivity. However, while different researchers express concerns about inter- and intra-observer error [[21], [22], [23]], few report on the extent to which these types of error affect the accuracy or reproducibility of the method being used, especially when it comes to the Mexican context.

The main aim of this study, then, is to determine the intra- and inter-observer agreement between five forensic experts in blind trials in order to assess whether reliability (intra-observer error) and reproducibility (inter-observer error) can significantly reduce errors in estimating the age of human remains, which would lead to more reliable personal identification. This can be achieved when several experts are available to work together as a team applying the same method.

## 2 Material and methods

### 2.1 Sample

The sample consisted of 96 X-ray images of teeth from 28 subjects (26 males and 2 females): 18 deceased people of Mexican origin (recovered from illegal burials) and 10 deceased migrants from Central America (7 from Guatemala and 3 from the Dominican Republic) who had been killed in car crash accidents (Table 1). All of the teeth collected were upper and lower healthy unaltered canines with fully formed roots (Table 2, Table 3), and all subjects were aged between 19 and 49 years at death. Each tooth had been extracted from the dental socket without damage, and all 96 X-ray images were analyzed by multidisciplinary post-mortem analysis units between 2020 and 2021.

**Table 1:** Sample distribution according to the circumstances of victims (fully identified), analyzed teeth, and nationality.

	Deceased	Analyzed teeth	Nationality
Illegal burials	18	66	Mexico
Migrants	10	30	Central America
Total	28	96

**Table 2:** Sample quantity of analyzed upper and lower canines.

	Quantity
Upper canines	49
Lower canines	47
Total	96

**Table 3:** Distribution of cases according to the presence of canine.

	Quantity
Deceased people with upper right canine	25
Deceased people with upper left canine	24
Deceased people with lower left canine	23
Deceased people with lower right canine	24
Total	96

Chronological age and country of origin/birth place were confirmed when the person was fully identified by one or more scientific methods for human identification. Age was calculated by subtracting date of birth from the date the subject went missing or died. Table 4 shows the distribution of the sample by age (categorized into groups: 19–29, 30–39, and 40–49 years) and sex.

**Table 4:** Age and sex distribution of the sample.

Sex	Age groups			Total
	19–29	30–39	40–49	
Male	16	5	5	26
Female	2	–	–	2
Total	18	5	5	28

**Fig. 1:**
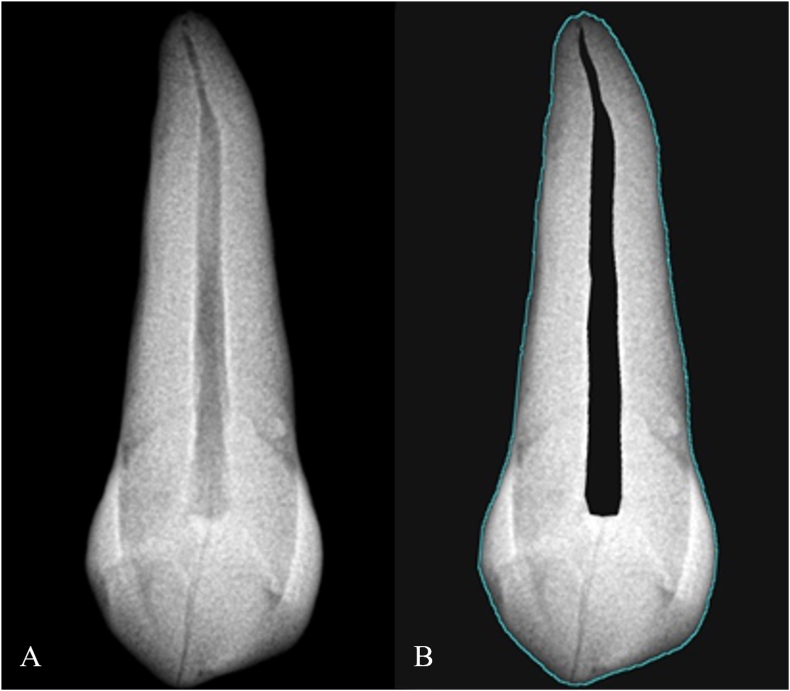
A digital system was used for periapical radiography. X-ray from extracted upper right canine (a); pulp and tooth outlines are well defined (b).

Digital periapical X-ray images were obtained in line with routine dental post-mortem analysis using Kodak Carestream RVG 5200 digital sensors and the Aribex Nomad Pro Dentalportable X-ray system (Fig. 1). Each tooth was placed in a vertical position no more than 5 cm from the digital receptor and exposed using the paralleling technique. The equipment was operated at 50 kV and 8 mA with an exposure time of 0.500 ms, and the two-digit notation system proposed by the FDI World Dental Federation (French: Fédération Dentaire Internationale) was adopted. The exclusion criteria were: visible third molar open apices, teeth with prosthetic restorations, any sign of endodontic treatment, and visible alterations such as wear or extensive cavities.

The five observers were of four different nationalities (Italian, Russian, Japanese, and Mexican) and each had over five years’ experience in the field of forensic anthropology or odontology. All observers were skilled at performing the age estimation method based on the pulp⁄tooth area ratio.

**Fig. 2:**
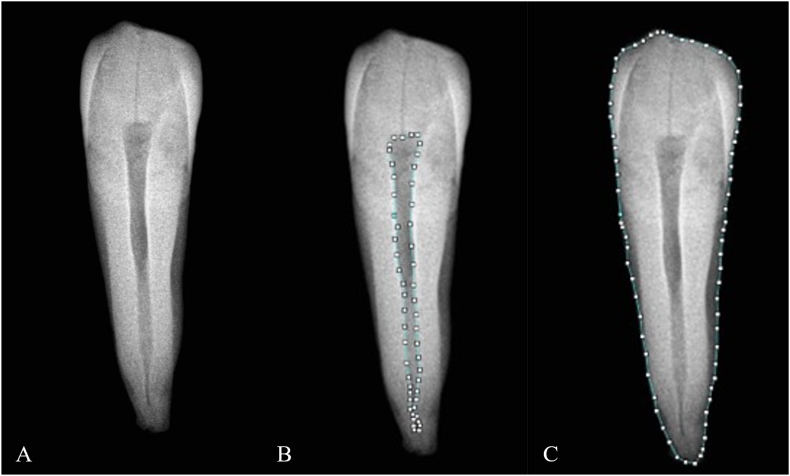
Digital image of a periapical X-ray from an extracted lower left canine (a); measurement of pulp area (b); measurement of tooth area (c). The polygon selection tool in ImageJ® software (National Institute of Health, Bethesda, Maryland, USA) was used [24].

A single forensic odontologist collected all dental information; the images were saved in high resolution JPEG digital format, and dental age assessment for each tooth was performed according to the pulp⁄tooth area ratio method [17]. To obtain the points of tooth and pulp outlines in order to evaluate tooth and pulp areas and area ratios, the ImageJ® public domain image processing program (National Institute of Health, Bethesda, Maryland, USA) was used [24] (Fig. 2).

The following morphological variables were recorded: TA = tooth area; PA = pulp area; date of death/disappearance; date of birth; sex; and nationality. Data were entered in a single Microsoft Excel© (Microsoft Corp., Redmond, WA, USA) file (used by all five observers). However, only the first observer (the person who collected the data of the whole sample) knew chronological age; the other four observers performed blind age-at-death estimations in order to test the intra- and inter-observer reliability [17].

The first observer assessed age at death for each of the 96 canines within 48 h of collecting the sample. The other four observers evaluated age at death one month later. Two months after the first results, the five observers evaluated twelve random cases from the twenty-eight deceased people, and the third and final evaluations were made by all five observers on the entire sample eight months after the first evaluations.

Cameriere et al.’s [17] linear regression equation was applied for each tooth according to upper or lower canine as follows:

Upper canine: Age = 99.937–532.775 * PA/TA.

Lower canine: Age = 89.456–461.873 * PA/TA.

### 2.2 Statistical analysis

The analysis was performed using the Statistical Package for the Social Sciences software (SPSS version 26.0, IBM Corporation, Armonk, New York, USA). Intra- and inter-observer agreement rates for the “age at death” variable were assessed with intraclass correlation coefficients using a two-way random method and expressed with 95% confidence. Thus, we evaluated absolute agreement between the 5 observers, as well as consistency of repeated measurements by the same observer at different times. The observers’ age-at-death estimates were compared to chronological age by linear regression, which generated the determination coefficient (R^2^), and error parameters were calculated. A significance level of 5% was adopted for the entire analysis (Table 5).

**Table 5:** Results obtained regarding intra- and inter-examiner agreement according to the intraclass correlation coefficient (ICC).

Agreement analysis type	Examiner/Exam	ICC (CI95%)	aClassification
Intra-examiner agreement	ST	0.953 (0.925–0.970)	Excellent
	A	0.798 (0.545–0.895)	Good
	SE	0.995 (0.993–0.997)	Excellent
	I	0.882 (0.811–0.926)	Excellent
	G	0.986 (0.975–0.992)	Excellent
Inter-examiner agreement	1st	0.975 (0.965–0.983)	Excellent
	2nd	0.927 (0.879–0.955)	Excellent

ICC = Intraclass correlation coefficient; CI95% = Confidence interval 95%.

a According to Landis & Koch [25].

## 3 Results

Both intra- and inter-observer agreement were considered satisfactory, in view of the good and excellent correlation coefficients (Table 5). The results indicate that the method under study could be reproduced with confidence both by the same operator at different times and by different evaluators.

For the agreement analysis, the data were evaluated using the two-way random method and absolute agreement was observed in the same evaluator at two different times (intra-examiner agreement), and between evaluators for each examination (inter-examiner agreement) [25].

**Fig. 3:**
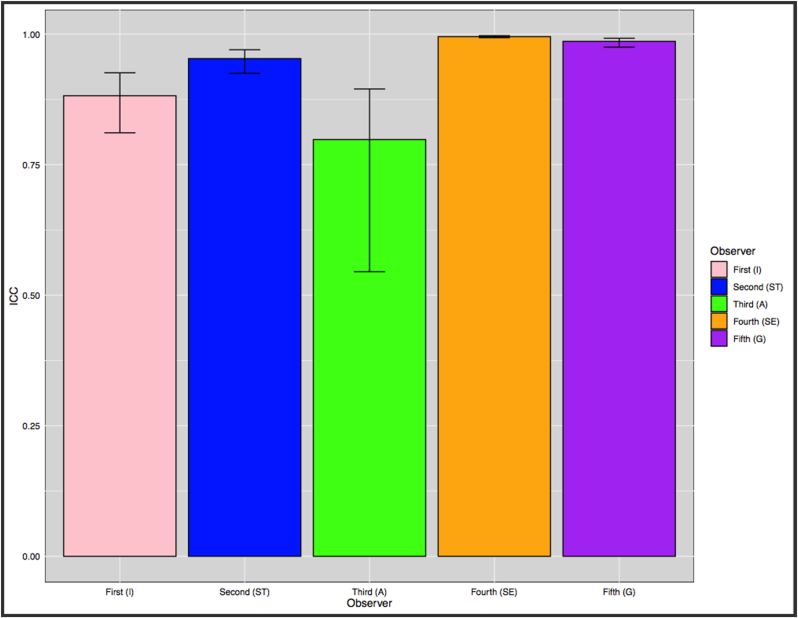
Results of the intra-class correlation coefficient (ICC) for the five examiners.

**Fig. 4:**
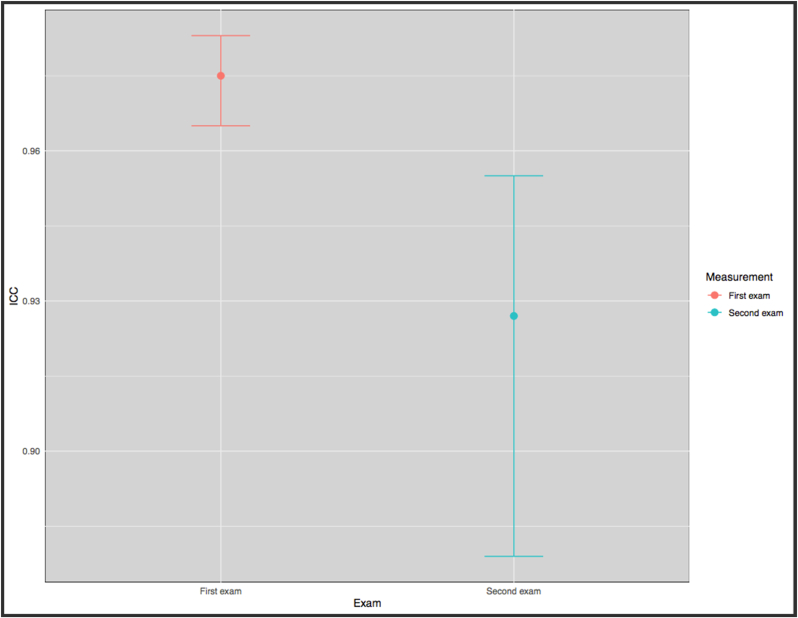
Plot of the inter-class correlation coefficient (ICC) of both exams.

The intraclass correlation coefficient values for four of the five observers showed high agreement between observers, indicating that the measurements were strong and reliable. In one evaluator only, agreement between evaluations was moderate. However, the results from all five observers indicated significant agreement on all measurements (Fig. 3, Fig. 4).

**Fig. 5:**
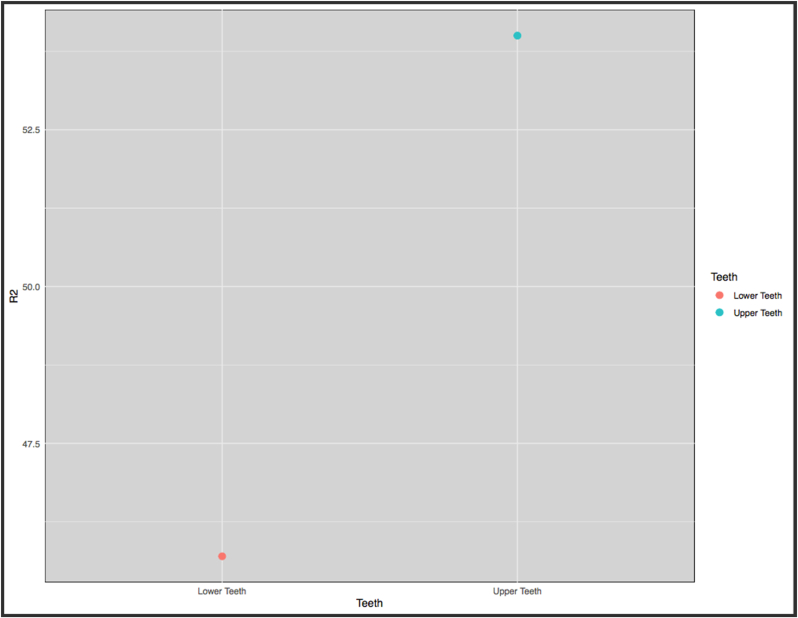
R^2^ values for upper and lower teeth.

Upper right canines provided a higher determination coefficient and lower mean prediction error compared to the lower right canines between all five observers. Therefore, upper canines offered more accurate age estimation with predictions closer to chronological age compared to the lower canines (Fig. 5).

The distribution of the difference between chronological and estimated age at death, according to observer and tooth evaluated for age-at-death estimation, also combining two and three teeth, are reported in Table 6, Table 7, respectively (Table 6, Table 7).

**Table 6:** Distribution of the difference between chronological and estimated age at death, according to observer and tooth evaluated for age estimation.

Observer	Tooth	R^2^	Mean chronological age (SD)	Mean estimated age (SD)	Mean difference (SD)	Median difference (IQR)	Mean prediction error (SD)
1	All	75.1%	29.16 (8.35)	30.74 (7.77)	−1.57 (4.12)	−2.87 (4.80)	0.49 (0.31)
	13	77.4%	29.76 (8.58)	31.04 (9.23)	−1.27 (4.32)	−1.72 (6.02)	0.49 (0.32)
	23	52.6%	28.93 (8.22)	30.22 (10.38)	−1.28 (7.01)	−0.80 (8.65)	0.69 (0.63)
	33	63.3%	28.75 (8.29)	31.35 (7,51)	−2.60 (4.97)	−3.31 (7.69)	0.61 (0.41)
	43	37.4%	28.43 (7.89)	28.97 (7.00)	−0.53 (6.43)	−0.47 (7.29)	0.62 (0.58)
2	All	67.9%	29.16 (8.35)	28.54 (8.21)	0.62 (4.81)	0.58 (3.79)	0.43 (0.47)
	13	72.7%	29.76 (8.58)	29.85 (8.53)	−0.08 (4.54)	0.79 (4.80)	0.42 (0.42)
	23	66.6%	28.93 (8.22)	28.95 (8.84)	−0.01 (5.08)	−0.12 (4.32)	0.43 (0.51)
	33	58.4%	28.75 (8.29)	28.89 (7.87)	−0.13 (5.41)	−0.37 (6.40)	0.50 (0.50)
	43	38.3%	28.43 (7.89)	26.71 (7.78)	1.71 (6.65)	1.84 (5.24)	0.61 (0.67)
3	All	68.1%	29.16 (8.35)	30.46 (7.93)	−1.29 (4.73)	−0.45 (1.64)	0.32 (0.56)
	13	69.5%	29.76 (8.58)	31.29 (8.90)	−1.52 (4.93)	−0.68 (2.83)	0.41 (0.54)
	23	56.8%	28.93 (8.22)	30.28 (8.72)	−1.35 (5.81)	−0.63 (1.57)	0.43 (0.66)
	33	69.7%	28.75 (8.29)	30.59 (7.42)	−1.84 (4.47)	−0.62 (2.96)	0.34 (0.54)
	43	42.7%	28.43 (7.89)	29.71 (7.81)	−1.28 (6.36)	−0.76 (3.13)	0.49 (0.70)
4	All	66.6%	29.16 (8.35)	28.09 (8.71)	1.07 (5.08)	0.51 (3.79)	0.44 (0.52)
	13	58.5%	29.76 (8.58)	28.65 (9.72)	1.11 (6.22)	2.22 (7.97)	0.65 (0.51)
	23	56.2%	28.93 (8.22)	27.95 (10.74)	0.98 (6.95)	0.92 (3.90)	0.61 (0.70)
	33	70.6%	28.75 (8.29)	28.44 (8.71)	0.30 (4.70)	1.09 (4.23)	0.44 (0.43)
	43	58.3%	28.43 (7.89)	27.01 (8.06)	1.42 (5.35)	2.22 (5.60)	0.56 (0.46)
5	All	45.0%	29.16 (8.35)	35.64 (10.31)	−6.48 (7.61)	−7.02 (13.80)	1.05 (0.80)
	13	38.8%	29.76 (8.58)	36.90 (11.24)	−7.13 (8.71)	−6.24 (11.53)	1.14 (0.96)
	23	38.2%	28.93 (8.22)	35.56 (10.97)	−6.62 (8.52)	−7.01 (13.48)	1.15 (0.84)
	33	37.8%	28.75 (8.29)	35.41 (9.87)	−6.65 (7.86)	−8.31 (11.92)	1.11 (0.79)
	43	26.6%	28.43 (7.89)	33.80 (9.62)	−5.36 (8.48)	−5.78 (15.04)	1.03 (0.84)
All	All	57.5%	29.16 (8.35)	30.69 (8.93)	−1.53 (5.97)	−0.66 (4.75)	0.55 (0.61)
	13	56.7%	29.76 (8.58)	31.54 (9.84)	−1.78 (6.52)	−0.68 (6.18)	0.62 (0.64)
	23	50.5%	28.93 (8.22)	30.59 (10.16)	−1.65 (7.16)	−0.30 (6.83)	0.66 (0.71)
	33	54.0%	28.75 (8.29)	30.94 (8.55)	−2.18 (6.05)	−0.93 (7.55)	0.60 (0.60)
	43	37.3%	28.43 (7.89)	29.24 (8.36)	−0.80 (7.09)	−0.29 (6.83)	0.66 (0.68)

R^2^ = Determination coefficient; SD: Standard deviation; IQR: Interquartile range. Linear regression. Significance level = 5%.

**Table 7:** Distribution of the difference between chronological and estimated age at death, according to combinations of two and three teeth evaluated for age-at-death estimation.

Teeth	R^2^	Mean chronological age (SD)	Mean estimated age (SD)	Mean difference (SD)	Median difference (IQR)	Mean prediction error (SD)
13 and 23	54.0%	29.36 (8.26)	31.08 (9.99)	−1.72 (6.83)	−0.44 (6.61)	0.64 (0.68)
13 and 33	55.8%	29.28 (8.29)	31.25 (9.23)	−1.97 (6.29)	−0.86 (6.92)	0.61 (0.62)
13 and 43	48.6%	29.11 (8.12)	30.42 (9.20)	−1.30 (6.81)	−0.48 (6.63)	0.64 (0.66)
23 and 33	52.0%	28.84 (8.09)	30.76 (9.39)	−1.91 (6.64)	−0.68 (7.04)	0.63 (0.66)
23 and 43	44.7%	28.68 (7.91)	29.92 (9.31)	−1.23 (7.13)	−0.29 (7.03)	0.66 (0.69)
33 and 43	45.7%	28.59 (7.93)	30.07 (8.48)	−1.48 (6.63)	−0.68 (7.37)	0.63 (0.64)
13, 23 and 33	53.9%	29.16 (8.21)	31.03 (9.54)	−1.86 (6.59)	−0.68 (6.84)	0.63 (0.66)
13, 23 and 43	49.3%	29.05 (8.10)	30.47 (9.51)	−1.42 (6.92)	−0.39 (6.77)	0.65 (0.68)
13, 33 and 43	50.1%	29.00 (8.12)	30.58 (8.99)	−1.58 (6.58)	−0.68 (6.85)	0.63 (0.64)
23, 33 and 43	47.4%	28.70 (7.97)	30.25 (9.07)	−1.54 (6.80)	−0.62 (7.00)	0.64 (0.67)

R^2^ = Determination coefficient; SD: Standard deviation, IQR: Interquartile range. Linear regression. Significance level = 5%.

## 4 Discussion

In contexts of missing and unidentified people, age-at-death estimates are an integral part of establishing the individual characteristics of the deceased person. Therefore, the proper age estimation method and criteria must be selected according to the age range.

Mexico is undergoing a forensic crisis regarding human identification. According to official numbers, over 52,000 unidentified bodies are being held in forensic facilities waiting to be fully identified, but this number could be higher [26].

In 2019, the state of Mexico approved the agreement and creation of the Extraordinary Forensic Identification Mechanism (MEIF) whose main purpose is to collaborate with the national authorities in the identification process of unidentified deceased people in the custody of forensic institutions. Once identified by valid scientific methods for human identification [27,28], the deceased must be handed over to their family members under decent conditions respecting human dignity in accordance with international human rights standards [29].

Mexico has constantly high rates of kidnappings, disappearances, and other criminal violence that has resulted in the deaths of tens of thousands of people in recent years [26]. Forensic anthropologists work with teams of forensic specialists that may include forensic pathologists, forensic odontologists, radiologists, fingerprint examiners, molecular biologists, mortuary technicians, photographers, and others.

Mass disasters are situations in which a multitude of victims need to be identified, and X-ray imaging is often a vital tool for victim identification. It is important to establish a team to carry out external examinations and autopsies, as well as instrumental examinations (especially dental) aimed at finding specific signs of identity; these teams also use radiographic examination tools to estimate sex and age [30].

In our experience, radiography has been widely used in post-mortem age estimation for the purposes of victim identification. Limiting the analysis to forensic odontology, several techniques have been developed to estimate chronological age in both children and adults, using the relationship between age and morphological changes in the structure of the teeth, particularly dimensional changes in the pulp area in relation to the tooth area [31].

Using a single tooth in age assessment can lead to errors in age estimation as the tooth under analysis might be the most worn down or not present in the mouth. Age assessment in forensic contexts requires access to as much of the sample as possible in order to perform individual analysis and obtain independent results per tooth to express a unique result. At least two canines are needed to perform Cameriere's age estimation method [32].

In the context of age assessment for forensic purposes, the importance of intra- and inter-observer reliability in the measurement of odontological parameters cannot be overstated [34]. Inter-observer reliability is defined as the agreement between two or more observers. Sometimes different observers produce different results because they do not use the measurement device in the same way [33]. Differences in readings could also be due to small random changes in the morphological variable itself during the measurement process. Intra-observer reliability, on the other hand, is defined as consistency of measurements by the same rater on two or more different occasions. Cohen's K statistic is commonly used for reliability assessments of categorical scales, while intraclass correlation coefficient (ICC) or concordance correlation coefficient (CCC) statistics are appropriate for continuous scales [35]. The greater the difference between the results, the poorer the inter- and intra-observer reliability of the survey [36].

In this case study sample, the deceased subject more often had four canines intact rather than just two. Since the linear regression equation model did not require any modification, the pulp⁄tooth area ratio method could be applied to the entire sample. In keeping with international forensic best practices, the method was validated by at least a second observer to confirm the results in age estimation [37].

A critical issue in the field of age estimation for forensic purposes is the interpretation of results. Combined anthropological methods are often used, but they give different results. Providing a court of law with a single result is not straightforward, given the risks of error involved in the application of different techniques. Our approach centers on a consolidated forensic method for obtaining a single value which is given by calculating the average of the measurements performed on multiple dental elements collected from the corpse; the specific standard deviation can be extrapolated from the same method. This certainly guarantees greater ease of interpretation of the data, and the results can be explicitly reported to a judicial authority.

### 4.1 Limitations

It is difficult to know which of the four permanent canines provides the best results in age-at-death estimation [34]. In comparison to third molars, canines are more susceptible to the effects of external factors such as dental wear, severe or low trauma, and even food and chewing frequently on one side. The forensic odontologist must be highly trained to confidently and accurately mark the precise points of pulp and tooth outlines in order to calculate pulp and tooth areas followed by pulp/tooth area ratios. At least two canines need to be evaluated for age assessment. For better results in pulp/tooth area ratios, dental X-rays should be obtained outside the tooth socket with digital sensors, along with image enhancement tools for better visibility. Another limitation of our study is the fact that the sample consisted predominantly of male subjects. However, the sex variable was considered in the analyses, and the validity of the study was demonstrated by low inter- and intra-operator variability using the method applied.

Finally, a limit of the study could be that of not having specific formulas available for the canine method in the populations of Guatemala or the Dominican Republic. However, this method of estimating age is not strictly dependent on ethnicity: possible variations, as found in some studies [32], could also depend on many other factors (nutrition, health, climate, etc.), which are unlikely to definable for each sample examined. The future challenge will be to apply the method on a large scale to evaluate ethnic differences, having to consider that a homogeneous sample, even within the same ethnic group, is difficult to obtain.

## 5 Conclusions

The pulp⁄tooth area ratio method is not undermined by subjectivity as it is a quantitative method that offers the advantage of sharing digital X-rays with other experienced observers around the world who can evaluate age with accuracy and precision if the sample is perfectly obtained applying the proper methodology. In contrast, in qualitative methods age can only be assessed by the person who took the sample or observed the changes in the evaluated teeth.

Mexico lacks validation in dental age estimation methods in contemporary samples from daily forensic cases. Therefore, the results obtained in the present case study are relevant for further research aimed at testing and verifying an age estimation method that does not require the use of complex or high-cost equipment for achieving results that are close to a person's chronological age.

Other forensic approaches where intra- and inter-operator variability were tested individually have not yielded better results than the method proposed in our study [38]. Forensic experts often resort to using different approaches where reliability is not often assessed. This is particularly true in subjects over the age of 40 where the anthropological and dental techniques commonly used give very unreliable results, often with very wide ranges.

## Funding

The authors did not receive support from any organization for the submitted work.

## CRediT authorship contribution statement

**Roberto Scendoni:** Writing – original draft, Conceptualization. **Israel Soriano Vázquez:** Writing – original draft, Conceptualization. **Isabella Lima Arrais Ribeiro:** Formal analysis. **Stefano De Luca:** Investigation. **Galina V. Zolotenkova:** Data curation. **Serena Viva:** Data curation. **Akiko Kumagai:** Validation. **Roberto Cameriere:** Supervision.

## Declaration of competing interest

The authors declare that they have no known competing financial interests or personal relationships that could have appeared to influence the work reported in this paper.
